# A Drive towards Thermodynamic Efficiency for Dissipative Structures in Chemical Reaction Networks

**DOI:** 10.3390/e23091115

**Published:** 2021-08-27

**Authors:** Kai Ueltzhöffer, Lancelot Da Costa, Daniela Cialfi, Karl Friston

**Affiliations:** 1 Wellcome Centre for Human Neuroimaging, Queen Square Institute of Neurology, University College London, London WC1N 3AR, UK; l.da-costa@imperial.ac.uk (L.D.C.); k.friston@ucl.ac.uk (K.F.); 2 Department of General Psychiatry, Center of Psychosocial Medicine, Heidelberg University, 69115 Heidelberg, Germany; 3 Department of Mathematics, Imperial College London, London SW7 2AZ, UK; 4 Department of Philosophical, Pedagogical and Economic-Quantitative Sciences, Economic and Quantitative Methods Section, University of Studies Gabriele d’Annunzio Chieti-Pescara, 65127 Pescara, Italy; daniela.cialfi@unich.it

**Keywords:** stochastic thermodynamics, dissipative structures, thermodynamic efficiency, chemical reaction networks

## Abstract

Dissipative accounts of structure formation show that the self-organisation of complex structures is thermodynamically favoured, whenever these structures dissipate free energy that could not be accessed otherwise. These structures therefore open transition channels for the state of the universe to move from a frustrated, metastable state to another metastable state of higher entropy. However, these accounts apply as well to relatively simple, dissipative systems, such as convection cells, hurricanes, candle flames, lightning strikes, or mechanical cracks, as they do to complex biological systems. Conversely, interesting computational properties—that characterize complex biological systems, such as efficient, predictive representations of environmental dynamics—can be linked to the thermodynamic efficiency of underlying physical processes. However, the potential mechanisms that underwrite the selection of dissipative structures with thermodynamically efficient subprocesses is not completely understood. We address these mechanisms by explaining how bifurcation-based, work-harvesting processes—required to sustain complex dissipative structures—might be driven towards thermodynamic efficiency. We first demonstrate a simple mechanism that leads to self-selection of efficient dissipative structures in a stochastic chemical reaction network, when the dissipated driving chemical potential difference is decreased. We then discuss how such a drive can emerge naturally in a hierarchy of self-similar dissipative structures, each feeding on the dissipative structures of a previous level, when moving away from the initial, driving disequilibrium.

## 1. Introduction

We start by briefly reviewing the role of dissipation in self-organisation on one hand, and the role of thermodynamic efficiency in the emergence of interesting computational properties—a hallmark of biological systems—on the other. We end by highlighting a small explanatory gap, namely how self-organising dissipative processes might be driven towards thermodynamic efficiency. We will then outline—through a heuristic argument substantiated by simulations of a stochastic chemical reaction network—a simple mechanism that pressures certain dissipative structures to become thermodynamically efficient.

Contrary to the—still widely held—belief that life is a struggle against the second law of thermodynamics, recent advances in nonequilibrium thermodynamics successfully recast biological systems as a subclass of dissipative structures. The formation of such dissipative structures is statistically favoured by (generalizations of) the second law of thermodynamics, because their existence enables the dissipation of reservoirs of free energy, which could not be accessed otherwise. Therefore, their formation facilitates the irreversible relaxation of the associated disequilibria [1,2,3,4,5]. In other words, dissipative structures constitute channels in the universe’s highly structured state space, which enable transitions from one frustrated, metastable state to another metastable state of higher entropy [6]. This line of thinking dates back at least to the work of Lotka, who tried to relate natural selection to a physical principle of maximum energy transformation [7,8]. Dissipative structure formation has been well understood for many systems in the near-equilibrium, linear-response regime, due to the work of Prigogine and colleagues in the 1960s and 1970s [9], leading to the notion of biological systems as a class of self-organising free energy-conversion engines [10]. However, it took several decades until thermodynamic equalities were derived that hold for small systems arbitrarily far from equilibrium. These equalities take the form of fluctuation theorems, which relate the entropy produced by a microscopic forward trajectory with the probability ratio of observing the forward, versus the time-reversed backward trajectory [11,12]. These fluctuation theorems generalized the relationship between entropy increase and irreversibility from the regime of macroscopic, closed systems at equilibrium—i.e., the second law of thermodynamics—to microscopic, open systems arbitrarily far from equilibrium. For introductory surveys of the resulting field of stochastic thermodynamics see, for example, [13,14], for a comprehensive review see [15]. Recently, dissipative self-organisation of macroscopic structures far from equilibrium was formalized through a macroscopic coarse-graining of such a microscopic fluctuation theorem [16]. This work relates the finite-time transition probabilities from one macroscopic state to two possible outcome states not only to the energy of the final states—via an equilibrium-like Boltzmann-term—and the kinetic barriers separating the initial from the potential finite states, but crucially also to the amount of dissipated heat during the transitions. Thus, energy levels and kinetic accessibility of potential final states being equal, outcome states with a higher dissipative history are favoured, leading to the coining of the term dissipative adaptation for such a selection process [16].

Taken together, this line of research provides a powerful account of how highly ordered nonequilibrium systems can emerge from basic thermodynamic principles, namely fluctuation theorems in stochastic thermodynamics and their special limiting case, the second law of thermodynamics (combined with an initial state of low entropy and a highly structured state space). However, in principle these accounts apply as well to relatively simple, dissipative systems, such as convection cells, hurricanes, candle flames, lightning strikes, or mechanical cracks, as they do to complex biological systems.

One important difference between life and non-life is the role of information processing in the ongoing physical processes [17,18,19]. Although simple dissipative systems, such as convection cells, are governed by thermodynamic constraints and fluxes flowing through them, living systems contain large amounts of information (e.g., stored in a cell’s DNA and epigenome), which—in stark contrast to simpler systems—govern and structure the thermodynamic fluxes through them. What kind of drives or pressures could facilitate the transition from a simple thermodynamic to an information-governed regime in dissipative systems? Two lines of work show that thermodynamic efficiency might play a role in this transition: In stochastic thermodynamics, the dissipated heat in a thermodynamic system, which is driven by a time-varying potential, upper bounds the system’s non-predictive information about the time-dependent drive [20]. Thus, to minimize the dissipated heat during the work extraction process, the system must develop an efficient, predictive representation of the driving environmental dynamics. From the requisite predictive dynamics, which can be linked to an information bottleneck [21], agent-like features, such as action policies that balance exploration and exploitation [22], and curiosity driven reinforcement-learning [23] can be derived (c.f., [24] for a synthesis). In computational neuroscience, adding energetic costs to the objective function minimized by simulated neural networks leads to the emergence of computational properties such as distributed neural codes, sparsity of representations, stochasticity, and the heterogeneity of neural populations [25,26,27,28,29,30,31,32] (c.f., [33] for a review).

It is still not fully understood, under which constraints thermodynamically efficient processes emerge during dissipative self-organisation. This work attempts to address this explanatory gap.

The results in this work are based on simulations and analyses of the dynamics and thermodynamics of stochastic chemical reaction networks. The application of stochastic thermodynamics to chemical reaction networks is a relatively young [34,35,36,37], and active field of research [38]. Recent works covered advances in energy-efficient dissipative chemical synthesis [39], and provided important extensions from homogeneous mixtures, ideal, and elementary chemical reactions, to spatially extended reaction-diffusion systems [40,41], non-ideal [42], and non-elementary chemical reaction networks [43]. Furthermore, thermodynamically consistent coarse-graining rules for chemical reaction networks were derived only recently [44]. These results might carry special importance for understanding large, hierarchical, and complex biochemical networks. Yet, in this work we call only on results concerning the nonequilibrium thermodynamics of spatially homogeneous mixtures, and ideal, elementary reactions in deterministic and stochastic chemical reaction networks [45,46].

The underlying idea is that in certain emergent, self-similar hierarchies of dissipative structures within a stochastic chemical reaction network, namely those without concentration processes, the available chemical driving potential decreases at each subsequent layer in the hierarchy, while the minimum work-rate required to sustain a new layer of dissipative structures might not. Thus, there might be a minimum viable efficiency of the thermodynamic processes, which must harvest the work required to maintain the associated dissipative structure from the conducted free energy fluxes. This pressure would increase with increasing hierarchical distance of the dissipative structures from the initial, driving disequilibrium.

To illustrate this, we construct chemical reaction networks, in which we explicitly implement instances of our proposed mechanisms: We start by demonstrating a simple mechanism that leads to a selection pressure on the thermodynamic efficiency of certain dissipative steady states in a stochastic chemical reaction network. The idea behind our simulations is simple: we construct a stochastic chemical reaction network, which may dissipate a large reservoir of chemical free energy via the maintenance of any one of a set of discrete, nonequilibrium steady states. Each of these nonequilibrium steady states is associated with the same, positive, finite minimum work-rate required to maintain it, and must maintain itself by harvesting the required work-rate from the conducted free energy flux via a bifurcation mechanism. Crucially, by making the thermodynamic efficiency of these work-harvesting processes different for each individual nonequilibrium steady state, a decrease of the dissipated driving chemical potential difference leads to a direct selection pressure on the nonequilibrium steady states. Indeed, only the corresponding bifurcation processes which are sufficiently efficient can sustain their associated nonequilibrium steady states.

We then discuss how this mechanism can lead to a selective pressure for thermodynamic efficiency in higher levels of a particular hierarchy of self-similar dissipative structures, based on the above network motif, where each dissipative structure is driven by (or feeding on) a dissipative structure at a lower level, i.e., closer to an initial, driving disequilibrium.

## 2. Materials and Methods

In this section, we explain the implementation details underlying our stochastic chemical reaction networks simulations. All calculations and simulations presented in this paper can be reproduced with code available at https://github.com/kaiu85/CRNs (accessed on 26 August 2021).

### 2.1. Stochastic Chemical Reaction Network Simulations

Most of our numerical experiments involving stochastic chemical reaction networks rest on sampling trajectories given a network architecture and initial network state. The state of a chemical reaction network, consisting of m chemical species $S_{1},\ldots ,S_{m},$ is described by the number n of molecules of each species S present in the reaction volume V. This yields the state vector $n=(n\left(S_{1}\right),\;n\left(S_{2}\right),\;\ldots ,n\left(S_{m}\right)$). The probability $p_{n}\left(t\right)$ of finding the network in the state ***n*** at a given time t evolves according to the chemical master equation

$$d_{t}p_{n}=\underset{\rho }{\sum }w_{-\rho }\left(n+S_{\rho }\right)p_{n+S_{\rho }}-w_{\rho }\left(n\right)p_{n}.$$

The sum is taken over individual forward reactions ρ, and −ρ denotes the corresponding backward reaction. $S_{\rho }$ represents the change in molecule numbers associated with the occurrence of the forward reaction ρ. Assuming elementary reactions and mass-action stochastic kinetics, we can write the reaction rates as

$$w_{\rho }\left(n\right)=k_{\rho }\frac{V}{\prod_{i}V^{\nu_{\rho }^{i}}}\frac{n!}{\left(n-\nu_{\rho }\right)!}.$$

Here $\nu_{\rho }=\left(\nu_{\rho }^{1},\nu_{\rho }^{2},\ldots ,\nu_{\rho }^{m}\right)$ is the vector of stochiometric coefficients of the reaction ρ. This definition ensures that the corresponding chemical reaction rate constants $k_{\rho }$ are the same as for the large volume limit considered in deterministic chemical kinetics [45,46]. 

To ensure that a closed stochastic chemical reaction network relaxes to a unique equilibrium steady state, therefore connecting dynamics to thermodynamics, we assume a local detailed balance relation between the rate constants of the forward and backward reaction directions

$$\ln\frac{k_{\rho }}{k_{-\rho }}=-\beta \mu^{0}\cdot S_{\rho }.$$

Here $\mu^{0}$ is the vector of standard-state chemical potentials, $\beta =\frac{1}{k_{B}T}$, $k_{B}$ denotes the Boltzmann constant, and T denotes the temperature of a large heat bath, which is in contact with the reaction network. A detailed discussion of the dynamics and thermodynamics of stochastic chemical reaction networks can be found in [46].

For simplicity, we set the volume V = 1, the standard chemical potentials $\mu^{0}=0$ for all species, corresponding to symmetric forward and backward rate constants $k_{\rho }=k_{-\rho }$, and use units of $k_{B}T=1$.

The assumption of an equal standard chemical potential $\mu_{S}^{0}=\mu^{0}=0$ for all species S effectively makes the equilibrium chemical potential a monotonous function of the species count n(S), and renders forward and backward reaction rate constants symmetric. This assumption furnishes an intuitive understanding of the dynamics along individual reactions as being driven by concentration gradients, i.e., by the tendency to relax towards a homogeneous equilibrium state. This allows intuitions from other homogeneous relaxation processes (e.g., heat diffusion in homogeneous media, water levels in pipeline systems) to carry over, as it aligns the direction of the driving concentration gradient with the direction of the driving chemical potential gradient. This assumption can easily be relaxed by choosing individual values of $\mu_{S}^{0}$ for each species. This will not affect our results or discussion on the self-selection of stable nonequilibrium steady states based on the efficiency of their work-harvesting processes, as long as one replaces “driving concentration gradient” with “driving chemical potential gradient”.

To sample individual trajectories for a given chemical reaction network and initial state, we implemented Gillespie’s stochastic simulation algorithm [47] based on Python 3 and PyTorch [48]. The latter allows us to use GPU-acceleration to parallelize over individual processes and chemical reactions.

### 2.2. Quantification of Minimum Work-Rate Required to Maintain Nonequilibrium Steady States

The minimum work-rate required to maintain a target nonequilibrium steady state equals the system’s heat production rate in the instant, when the forcing maintaining the nonequilibrium steady state is stopped, i.e., when the system is closed and just begins to relax to equilibrium [49]. Therefore, we estimated the minimum work-rate required to maintain a given nonequilibrium steady state with the following approach: We simulated an ensemble of $10^{5}$ stochastic trajectories at a target nonequilibrium steady state, by initializing the trajectories close to that particular nonequilibrium steady state, and stabilizing that state by opening the chemical reaction network via corresponding chemostatted species [46]—i.e., allowing for external nonequilibrium forces. We then closed the system, by releasing the clamps from the chemostatted species, treating them as any other species of the network henceforth. Therefore, the closed network immediately started to relax to its equilibrium steady state. Recall that the heat rate dissipated just at the beginning of this relaxation process equals the minimum work-rate required to maintain the corresponding nonequilibrium steady state [49]. Thus, we simulated ten additional time steps (i.e., reaction events) for each trajectory just after the system was closed. We used the time to Δt and free energy Δg released by the occurring reaction event to calculate the instantaneous heat dissipation rate via averaging the fraction $\frac{\Delta g}{\Delta t}=\frac{g\left(n\left(t_{10}\right)\right)-g\left(n\left(t_{0}\right)\right)}{t_{10}-t_{0}}$ over trajectories. Here $t_{0}$ is the time when the network was closed, $t_{10}$ is the time of occurrence of the tenth reaction event thereafter,$\;g\left(n(t_{0}\right))$ is the initial Gibbs free energy just when the network was closed, and $g\left(n\left(t_{10}\right)\right)$ is the Gibbs free energy of the closed network after the tenth reaction event. We quantified the Gibbs free energy g of the individual, closed chemical reaction network states using $g\left(n\right)=kT\underset{S}{\sum }\ln\left(n\left(S\right)!\right)$, following [46]. The sum is taken over all chemical species S, and n(S) is the number of molecules of chemical species S. This formula reflects our choice of the same chemical standard potential of $\mu_{0}=0$ for all species.

### 2.3. Quantification of the Thermodynamic Efficiency of Bifurcation-Based Work-Harvesting Processes

To quantify the average efficiency of different nonequilibrium steady states, we used the deterministic rate equations for the corresponding, large chemical reaction network (i.e., in the limit $n\left(S\right)\to \infty ,\;V\to \infty ,\frac{n\left(S\right)}{V}=c\left(S\right)=\mathrm{const}.$ for species counts n(S) and reaction volume V), and calculated the fluxes and the thermodynamic forces along each forward-backward pair of reactions, following [45]. This allowed us to calculate the heat dissipated along each forward-backward reaction pair via

$${\dot{Q}}_{\rho }=-{\Delta G}_{\rho }J_{\rho }.$$

Here $J_{\rho }=J_{\rho }^{+}-J_{\rho }^{-}$ is the net flux, i.e., the difference between the fluxes $J_{\rho }^{+}$ in the forward and $J_{\rho }^{-}$ in the backward reaction direction. The thermodynamic forces ${\Delta G}_{\rho }$ are calculated as the chemical potential difference along the forward direction via

$${\Delta G}_{\rho }=\underset{p\in \mathrm{products},\rho^{+}}{\sum }\mu_{p}-\underset{e\in \mathrm{educts},\rho^{+}}{\sum }\mu_{e}.$$

As before, we are using $\mu_{0}=0$ for all species, reducing the nonequilibrium chemical potentials to $\mu_{\sigma }=RT\ln\frac{Z_{\sigma }}{Z_{\sigma }^{\mathrm{eq}}}$, where $Z_{\sigma }$ is the concentration of species σ, and $Z_{\sigma }^{\mathrm{eq}}$ is the corresponding equilibrium concentration.

## 3. Results

In the following sections, we first generalize the bistable Schlögl network [50] to multiple, competing chemical species, leading to winner-take-all dynamics and multistability. We then show that to maintain any of the high-concentration steady states, a finite, minimum work-rate is required. In our simulations, this corresponds to a finite, positive driving chemical potential difference, which—due to our choice of $\mu^{0}=0$ for the standard chemical potential of all chemical species—means just a finite, positive driving concentration gradient.

Using this winner-take-all motif as a chemical switch, we then integrate it into a larger network, where the dissipation of a concentration gradient is dependent on the presence of a high-concentration steady state of the winner-take-all network. At the same time, the winner-take-all network must harvest the work (or corresponding concentration gradient) to sustain itself directly from the conducted free energy flux via a bifurcation mechanism. By ensuring the efficiency of these bifurcation mechanisms differs for each of the high-concentration states, we see that a reduction of the driving concentration gradient leads to a strong selection pressure on the corresponding nonequilibrium steady states: Only the states with bifurcation processes of sufficient thermodynamic efficiency can retain their stability, when the driving concentration gradient is decreased.

In the discussion section, we will discuss how in a self-similar hierarchy of dissipative systems such a pressure might develop spontaneously, due to a decrease of the available, driving concentration gradient at each successive layer of dissipative structures.

### 3.1. Chemical Winner-Take-All Dynamics

**Figure 1:**
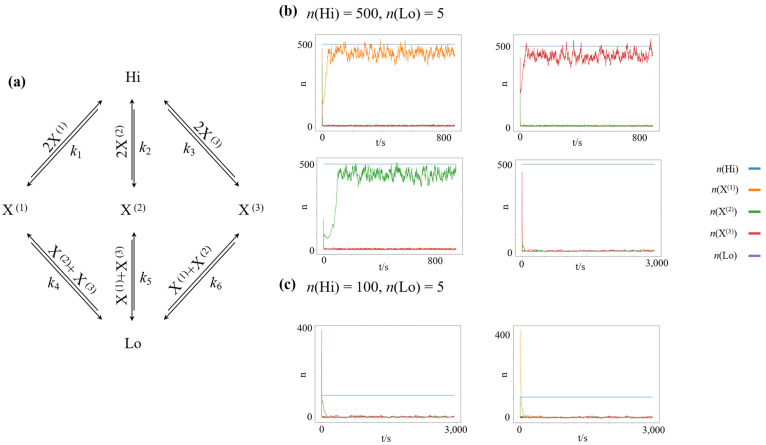
Nonlinear chemical reaction network, featuring winner-take-all attractor dynamics. Crucially, all three high-concentration nonequilibrium steady states are symmetric with respect to exchanging X^(1)^, X^(2)^, and X^(3)^. Thus, the associated minimum work-rate required (and the associated minimum driving concentration gradient n(Hi)−n(Lo) to maintain each of these states is the same. (**a**) Layout of the chemical reaction network (**b**) Simulations of randomly initialized networks, with driving species clamped at n(Hi)=500, n(Lo)=5. Given this forcing, high-concentration states of an individual species $X^{\left(1\right)}$, $X^{\left(2\right)}$ or $X^{\left(3\right)}$, as well as a low-concentration state are stable attractors of the dynamics. (**c**) Simulations of randomly initialized networks, with driving species clamped at n(Hi)=100, n(Lo)=5. Given this forcing, only the low-concentration state constitutes an attractor of the dynamics.

The architecture of a chemical reaction network featuring winner-take-all dynamics is shown in Figure 1a. It is a straightforward extension of the nonlinear chemical reaction network introduced by Schlögl [50], which is the simplest bistable reaction network. The network is driven out of equilibrium by a fixed concentration difference between two chemostatted species, Hi and Lo, with n(Hi)≫n(Lo). We chose the equilibrium potential $\mu^{0}=0$ for all species in our simulation, effectively rendering all reaction channels symmetric. Thus, the closed chemical reaction network would relax to an equilibrium distribution with equal counts $n_{eq}$ for all species. To allow for nonlinear behavior, we introduce three dynamic chemical species, $X^{\left(1\right)}$,$\;X^{\left(2\right)}$, and $X^{\left(3\right)}$, which autocatalyze their own creation from the high-concentration species Hi. Furthermore, to introduce inhibitory competition between the dynamic species, we introduce decay reactions to the low-concentration species Lo for each dynamic species, which are catalysed by the competing species. Using the reaction rate constants $k_{1}=k_{2}=k_{3}=10^{-6}$ and $k_{4}=k_{5}=k_{6}=10^{-3}$, and the fixed concentrations n(Hi)=500 and n(Lo)=5 the resulting network features four stable attractors: One symmetric, low-concentration steady state, where $n\left(X^{\left(1\right)}\right)\approx n\left(X^{\left(2\right)}\right)\approx n\left(X^{\left(3\right)}\right)\approx n\left(\mathrm{Lo}\right)$, and three high-concentration steady states of species $X^{\left(1\right)}$, namely $n\left(X^{\left(1\right)}\right)\approx n\left(\mathrm{Hi}\right),\;n\left(X^{\left(2\right)}\right)\approx n\left(X^{\left(3\right)}\right)\approx n\left(\mathrm{Lo}\right)$, species $X^{\left(2\right)}$, namely $n\left(X^{\left(2\right)}\right)\approx n\left(\mathrm{Hi}\right),\;n\left(X^{\left(3\right)}\right)\approx n\left(X^{\left(1\right)}\right)\approx n\left(\mathrm{Lo}\right)$; and species $X^{\left(3\right)}$, namely $n\left(X^{\left(3\right)}\right)\approx n\left(\mathrm{Hi}\right),\;n\left(X^{\left(1\right)}\right)\approx n\left(X^{\left(2\right)}\right)\approx n\left(\mathrm{Lo}\right)$, as shown in Figure 1b.

Using the same reaction rate constants but decreasing the concentration difference to n(Hi)=100 and n(Lo)=5, the high-concentration states lose their stability, and the only remaining stable attractor is the symmetric low-concentration state, as shown in Figure 1c.

### 3.2. Finite Minimum Work-Rate Required to Maintain High-Concentration States

To quantify the range of stability of high-concentration steady states, we run a series of simulations initializing the network close to the high-concentration steady state of species X^(1)^, i.e., $n\left(X^{\left(1\right)}\right)=n\left(\mathrm{Hi}\right),\;n\left(X^{\left(2\right)}\right)=n\left(X^{\left(3\right)}\right)=n\left(\mathrm{Lo}\right)$, and simulating $10^{5}$ processes for 300,000 reaction steps. When the ensemble of trajectories has converged to a high-concentration steady state, we save the network state and calculate the minimum work-rate required to maintain the high-concentration steady state (c.f., methods section). As the network architecture is completely symmetric with respect to permutations of $X^{\left(1\right)}$,$\;X^{\left(2\right)}$, and $X^{\left(3\right)}$, the resulting minimum work-rate is the same also for high-concentration steady states of species $X^{\left(2\right)}$ and $X^{\left(3\right)}$.

**Figure 2:**
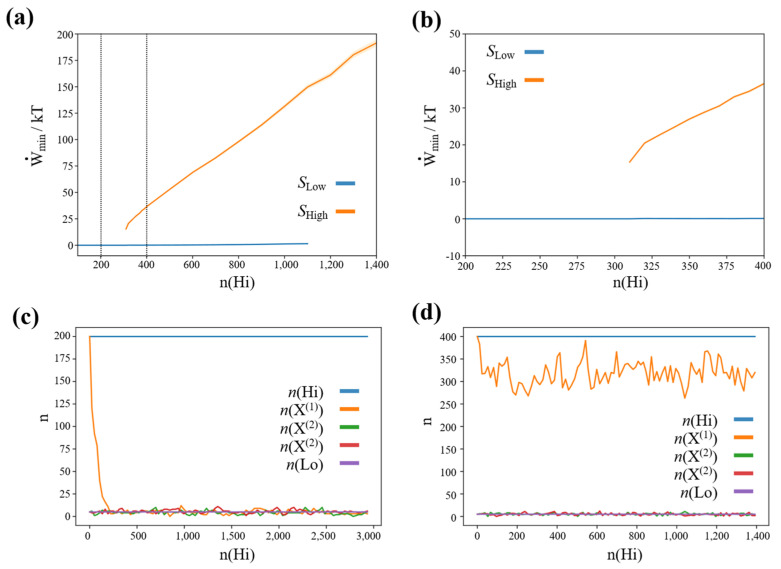
(**a**,**b**) Minimum work-rate (equivalent to a minimum concentration difference n(Hi)−n(Lo), where n(Lo)=5=const.) required to maintain stable nonequilibrium, high-concentration states in a stochastic chemical reaction network featuring winner-take-all dynamics. $S_{\mathrm{Low}}$: Low-concentration steady state, $S_{\mathrm{High}}$: High-concentration steady state. (**c**) Representative simulation trajectory for n(Hi)=200. (**d**) Representative simulation trajectory for n(Hi)=400.

We also performed a series of simulations, initializing the network close to the low-concentration steady state i.e., $n\left(X^{\left(1\right)}\right)=n\left(X^{\left(2\right)}\right)=n\left(X^{\left(3\right)}\right)=n\left(\mathrm{Lo}\right)$, using the same procedure and parameters. The resulting plot of the stability of the high- and low-concentration steady states, and the associated minimum work-rate required is shown in Figure 2a,b.

We see that below a critical concentration of the high-concentration species (and therefore below a critical driving concentration difference) of $n_{crit}\left(\mathrm{Hi}\right)\approx 310$ the high-concentration states lose their stability. Furthermore, we see that there is a positive, finite minimum work-rate associated with this concentration gradient.

Thus, in summary, the stability of high-concentration steady states in our winner-take-all chemical reaction network requires a positive, finite minimum work-rate (and associated concentration gradient). Furthermore, by construction this work-rate is the same for all high-concentration steady states.

### 3.3. Decreasing the Driving Chemical Potential Difference Leads to Self-Selection of Nonequilibrium Steady States with High-Efficiency Bifurcation Processes

**Figure 3:**
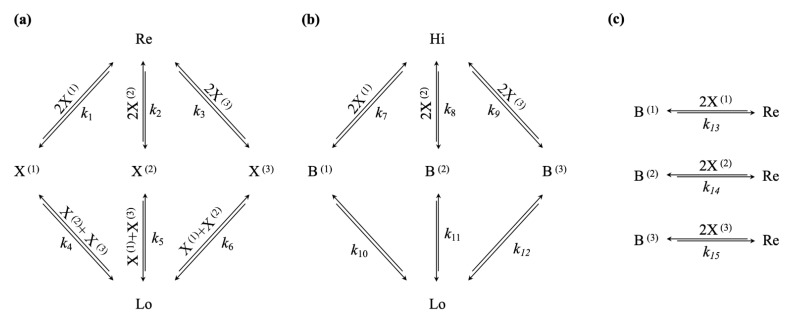
(**a**) Multi-stable winner-take-all network, featuring a single low- and three high-concentration nonequilibrium steady states, given a sufficiently high-concentration of reservoir species Re; (**b**) Disequilibrium/Free energy reservoir given by the concentration gradient n(Hi)−n(Lo), the dissipation of which is conditional on a high-concentration steady state of the winner-take-all network, by means of the catalysed reaction channels from Hi to B^(1)^, B^(2)^, or B^(3)^; (**c**) Bifurcation mechanisms, coupling the dissipative channel to the maintenance of high-concentration states in the winner-take-all network, via the conversion of B^(1)^, B^(2)^, or B^(3)^ to the reservoir species Re.

We now can construct the chemical reaction network evincing the selection mechanism that we want to discuss in this paper: We use the same winner-take-all attractor motif; however, we do not directly connect $X^{\left(1\right)}$,$\;X^{\left(2\right)}$, and $X^{\left(3\right)}$ to the clamped high-concentration species Hi, but rather to a dynamic reservoir species Re, as shown in Figure 3a. Next, we introduce three potential relaxation channels, allowing the conversion of a molecule of the clamped, high-concentration species Hi to a molecule of the clamped low-concentration species Lo via three possible intermediate species, $B^{\left(1\right)}$,$\;B^{\left(2\right)}$, and $B^{\left(3\right)}$. Crucially, we make the synthesis of these intermediary species dependent on catalysis by two molecules of $X^{\left(1\right)}$,$\;X^{\left(2\right)}$, or $X^{\left(3\right)}$, respectively, as shown in Figure 3b. Finally, we couple the maintenance of the high-concentration winner-take-all attractor states to the conducted free energy flow, via a bifurcation process, turning a molecule of $B^{\left(1\right)}$,$\;B^{\left(2\right)}$, or $B^{\left(3\right)}$ into a molecule of the reservoir species Re. Again, we make these reactions depending on two molecules of $X^{\left(1\right)}$,$\;X^{\left(2\right)}$, or $X^{\left(3\right)}$, respectively, as shown in Figure 3c.

**Figure 4:**
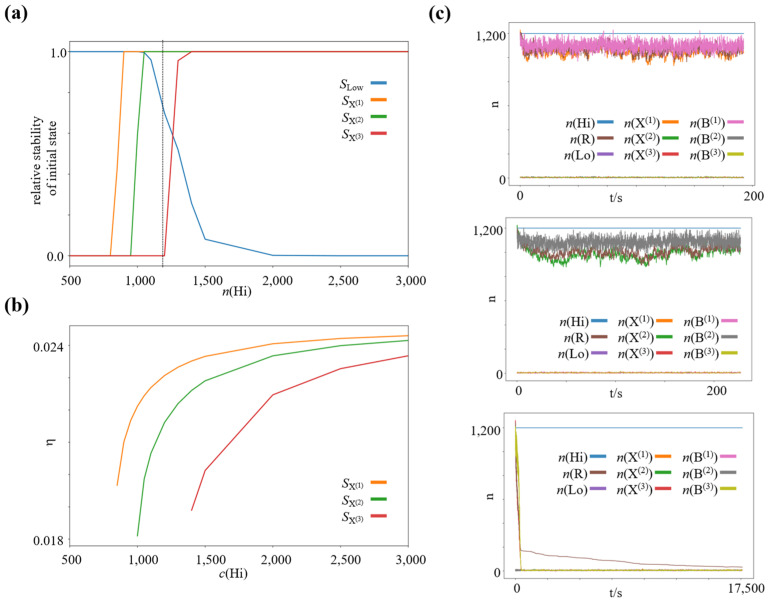
Increasing selection pressure on the efficiency of the work-harvesting processes maintaining the dissipative nonequilibrium steady states with decreasing available chemical potential difference. (**a**) Relative stability of the low and high-concentration steady states as function of n(Hi), where n(Lo)=5=const. (**b**) Efficiency η of the bifurcation processes at the individual high-concentration nonequilibrium steady states of species X^(1)^, X^(2)^, and X^(3)^ as function of c(Hi), where c(Lo)=5=const. (**c**) Representative trajectories of a chemical reaction network initialized close to each high-concentration steady state for n(Hi)=1200. $S_{X^{\left(1\right)}}$: high-concentration state of species X^(1)^, $S_{X^{\left(2\right)}}$: high-concentration state of species X^(2)^, $S_{X^{\left(3\right)}}$: high-concentration state of species X^(3)^, $S_{\mathrm{Low}}$: Low-concentration steady state.

We run a series of simulations for a wide range of concentration gradients, by varying the concentration of the high-concentration species n(Hi), while keeping n(Lo)=5 constant. We use the same rate constants as before for the winner-take-all subnetwork, namely $k_{1}=k_{2}=k_{3}=10^{-6}$ and $k_{4}=k_{5}=k_{6}=10^{-3}$. We set the other reaction rate constants to $k_{7}=k_{8}=k_{9}=10^{-5}$ and $k_{10}=k_{11}=k_{12}=1.0$. We couple the intermediate species $B^{\left(1\right)}$,$\;B^{\left(2\right)}$, or $B^{\left(3\right)}$ to the reservoir species Re by different reaction rates, $k_{13}=10^{-6}$, $k_{14}=3\cdot 10^{-7}$ and $k_{15}=10^{-7}$, which leads to different efficiencies of the associated bifurcation process. We simulate 4000 processes, 1000 of which we initialize close to each of the four potentially stable states of the winner-take-all subnetwork. Specifically, 1000 close to the low-concentration state, n(X(1))=n(X(2))=n(X(3))=n(B(1))=n(B(2))=n(B(3))=n(Re)=n(Lo), 1000 close to the high-concentration state of species $X^{\left(1\right)},$ n(X(1))=n(B(1))=n(Re)=n(Hi), n(X(2))=n(X(3))=n(B(2))=n(B(3))=n(Lo), 1000 close to the high-concentration state of species $X^{\left(2\right)},$ n(X(2))=n(B(2))=n(Re)=n(Hi), n(X(3))=n(X(1))=n(B(3))=n(B(1))=n(Lo), and 1000 close to the high-concentration state of species $X^{\left(3\right)},$ n(X(3))=n(B(3))=n(Re)=n(Hi), n(X(1))=n(X(2))=n(B(1))=n(B(2))=n(Lo). We simulated 6,000,000 reaction steps for each process and checked if it had converged to the corresponding steady state. For each external forcing, we quantified the relative number of processes that converged to the steady state, which they were initialized close to, as a proxy for this state’s stability. The resulting graphs are shown in Figure 4a. As expected, for a very strong driving concentration gradient, all possible high-concentration states are stable (and the low-concentration state actually loses its stability), in analogy to the Schlögl model [50]. However, as the driving concentration gradient is decreased, more and more high-concentration states lose their stability. The first state to lose its stability is the high-concentration state of species $X^{\left(3\right)}$, which features the bifurcation (i.e., work-harvesting) process of lowest efficiency. When decreasing the driving concentration gradient further, the next high-concentration state to lose its stability is the high-concentration state of species $X^{\left(2\right)}$, featuring the bifurcation process of second lowest efficiency. When we reduce the driving concentration gradient even further, the high-concentration state of species $X^{\left(1\right)}$, featuring the most efficient bifurcation process, also loses its stability.

The average thermodynamic efficiency of the bifurcation processes at the individual high-concentration steady states was computed using the deterministic limit of the stochastic chemical reaction network, i.e., taking $n\left(S\right)\to \infty ,\;V\to \infty ,\frac{n\left(S\right)}{V}=c\left(S\right)=\mathrm{const}.$ Using the established theory of thermodynamics in deterministic, open chemical reaction networks ([45]; c.f., Methods), we calculated the average total dissipated heat ${\dot{Q}}_{\mathrm{tot}}$ in the entire network, and the average heat ${\dot{Q}}_{\mathrm{diss}}$ dissipated along the direct decay pathways from Hi via $B^{\left(1\right)}$,$\;B^{\left(2\right)}$, and $B^{\left(3\right)}$ to Lo at each high-concentration steady state. At steady state, the total dissipated heat corresponds to the total chemical work put into the network by the driving potential gradient (see for example [45,46]), thus we can calculate ${\dot{Q}}_{\mathrm{tot}}-{\dot{Q}}_{\mathrm{diss}}={\dot{W}}_{\mathrm{chem},\mathrm{tot}}-{\dot{Q}}_{\mathrm{diss}}={\dot{W}}_{\mathrm{chem},\mathrm{WTA}}$, which yields the chemical work, which is bifurcated to maintain the high-concentration state of the winner-take-all module (or equivalently, the chemical work required to pay the corresponding house-keeping heat). Now we can calculate the associated efficiency via $\eta =\frac{{\dot{W}}_{\mathrm{chem},\mathrm{WTA}}}{{\dot{W}}_{\mathrm{chem},\mathrm{tot}}}$. We plotted the corresponding efficiencies for the high-concentration steady states in Figure 4b. In this particular setup, all three processes are highly inefficient; however, the small absolute efficiency difference is sufficient to create strong, specific selection pressures on the corresponding nonequilibrium steady states when the driving concentration gradient is decreased.

## 4. Discussion

### 4.1. A Simple Selection Mechanism for Thermodynamic Efficiency in Dissipative Nonequilibrium Steady States of Chemical Reaction Networks

This work demonstrates a relatively simple mechanism, which leads to selective pressure on the efficiency of bifurcation-based work-harvesting processes in dissipative nonequilibrium steady states—following a decrease in the dissipated, driving disequilibrium. It rests on conditioning dissipative fluxes—along the driving concentration gradient in an open chemical reaction network—on a high-concentration state in a winner-take-all chemical reaction network. In turn, the winner-take-all network must harvest the work required to maintain this state from the very free energy fluxes that it enables, via a bifurcation mechanism. 

Although it would be conceivable in principle that the dissipative structure was fueled by a different energy source than the one whose dissipation it facilitated, recent work showed that the molecular level free energy-conversion processes, which living systems use to harvest the work to maintain their own structure, must rely on such turnstile-, bifurcation- or escapement-like mechanisms [1,51]. Furthermore, the fact that at least some of the associated molecular engines—such as the F1-ATPase [52]—work very close to optimal efficiency, might be a hint that selection pressures towards thermodynamic efficiency might have played a role in the development of these structures.

Furthermore, while we only discuss a very specific special case here, based on simplifying assumptions such as symmetric reaction rate constants for forward and backward reaction directions, the general premise—namely that dissipative structures require a positive, finite minimum work-rate to persist, and that they have to harvest this work directly from the conducted dissipative free energy fluxes, therefore requiring a specific minimum efficiency of the associated work-harvesting processes—should be applicable to a wider class of dissipative structures.

### 4.2. Emergent Pressure towards Thermodynamic Efficiency in a Hierarchy of Dissipative Structures

**Figure 5:**
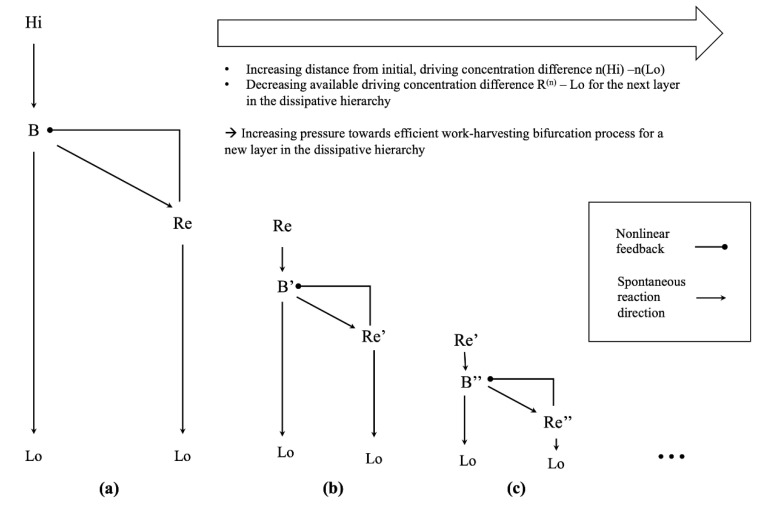
(**a**) Schematic depiction of the chemical reaction network discussed in this paper. The nonlinear feedback between the reservoir species Re and the intermediate species B in our simulation is mediated by the dynamics of the winner-take-all module. (**b**) It is conceivable that a similar dissipative structure develops, which does not feed on the initial driving concentration gradient n(Hi)−n(Lo), but on the structure realizing the dissipative process, in terms of the concentration gradient n(Re)−n(Lo) between the reservoir species of the initial dissipative structure and the low-concentration species. (**c**) This process could be iterated, by imagining a dissipative structure which now feeds on the reservoir species of the second order dissipative structure. One immediately sees that the concentration gradient available to drive the next layer in such a hierarchy of dissipative structures decreases the further one moves away from the initial driving concentration gradient.

Although we manually decreased the driving concentration difference in our simulations, one could easily imagine a similar pressure arising naturally, for example in an emergent, self-similar hierarchy of dissipative structures, each feeding on—i.e., dissipating—a lower level of dissipative structures (cf. [1,2]), in the following way: 

The steady state concentration gradient n(Re)−n(Lo), which is required to sustain the dissipative fluxes between Hi and Lo in our simulated network, constitutes a disequilibrium by itself. This disequilibrium can drive the emergence of another layer of dissipative systems with a similar structure. For example, one could just add another winner-take-all dissipation motif in a hierarchical fashion, as shown in Figure 5. Due to the construction of our reaction network, the concentration gradient n(Re)−n(Lo) and the associated chemical potential difference will be smaller than that of the initial, driving disequilibrium n(Hi)−n(Lo). Furthermore, assuming a similar architecture of the “higher order” winner-take-all network, including the reaction rate constants, the minimum work-rate required to maintain the corresponding nonequilibrium steady state will be similar to that of the original dissipative structure. Thus, there is already a slight increase in the minimum efficiency of the new bifurcation process required to harvest enough work to maintain the additional layer of dissipative structures. Given a sufficiently complex chemical space, one might imagine another layer of dissipative processes, feeding on the structure afforded by the second order winner-take-all network, e.g., in terms of the concentration gradient n(Re′)−n(Lo), where n(Re′)−n(Lo)<n(Re)−n(Lo)<n(Hi)−n(Lo). Thus, by iterating this argument over additional, self-similar layers of higher order dissipative structures—each feeding on the structures afforded by the previous layer of dissipative structures—an increasing pressure towards efficient bifurcation processes would develop as one moved further away from the initial, driving disequilibrium in this dissipative hierarchy. In this setting, a new layer of dissipative structure could only form if the associated work-harvesting bifurcation process were efficient enough to sustain it. As the available driving concentration gradient would monotonically decrease with each new layer of dissipative structures, but the minimum work required to sustain each successive layer would not, one could expect to find highly efficient, close to optimal dissipative structures in higher levels of this dissipative hierarchy—given a sufficiently complex chemical space and enough time for the chemical network to discover these highly efficient nonequilibrium steady states.

In a more realistic setting, variations in the minimum work-rate, required to form a new layer of dissipative structures, might lead to a non-monotonic relationship between the distance of a layer of dissipative structure from the initial driving disequilibrium, and the minimum efficiency required by the associated work-harvesting process to sustain this new layer of dissipative structures. However, while the available driving chemical potential difference will in general decrease with the hierarchical level of dissipative processes in the absence of concentration processes (i.e., processes which can turn two or more molecules of low chemical potential into a molecule of higher chemical potential), the minimum work-rate required to maintain a new layer of dissipative structure does not have to: it might decrease, stay constant, or increase. Thus, it does not seem too implausible that—in many chemical systems—the required work-rate to sustain a new layer of dissipative structure might come close to the maximum available free energy flux, therefore leading to a correspondingly high minimum efficiency required by the associated work-harvesting process. However, there are many processes in biology, for which this argument does not hold. Prominent counterexamples are food webs, in which plants concentrate the free energy of low entropy photons, e.g., in terms of vegetable fats and carbohydrates. These are then further concentrated by herbivores, e.g., in terms of animal fats and protein, which provide carnivores with a source of highly concentrated chemical free energy (for a discussion of adaptive dynamics and potential self-similarity in food webs, see [53]).

Although our work demonstrates self-selection of a subset of stable nonequilibrium steady states—namely those associated with sufficiently efficient work-harvesting processes to maintain themselves—we do not address the question of how probable different trajectories leading towards these states might be. This is an interesting question as recent work shows that the probability of arriving at a given nonequilibrium steady state is dominated by the dissipative histories of the trajectories leading there [54,55], which in general cannot not be related directly to the dissipation at the steady state itself. However, we can recover a qualitative relationship between our results and the general notion of dissipative adaptation from the following, heuristic argument: In real systems, the dissipated concentration gradient would feed from a finite reservoir, i.e., disequilibrium, which must be dissipated at some point to allow for the whole—initially highly frustrated—system to relax to equilibrium. In our case, the relative probability of that reservoir being dissipated without realizing one of the stable high-concentration nonequilibrium steady states is, by construction, virtually zero. Using our simulation framework to quantify the relative probabilities of reaching equilibrium via the available, self-selected nonequilibrium steady states, might be an interesting avenue for future research.

## 5. Conclusions

Given the large and high-dimensional, yet highly structured (in the sense that not every chemical reaction is allowed) nature of chemical space, leading to intriguing—and only partly understood—structures and dynamics in the most complex chemical reaction networks we know, namely those in biochemistry (e.g., [17,56,57]), it seems reasonable to assume that similar dissipative hierarchies and associated pressures towards thermodynamic efficiency might have emerged in biological systems. This may be the case, as existing biogeochemical networks feature a universal scaling law, which is not a product of the underlying chemical space alone [56]. The mechanisms considered in this work might therefore constitute one of many drives leading to the transition of early biochemistry from a thermodynamic- to an information-determined regime, which is considered a hallmark of the living state [18,19].

## Data Availability

Code reproducing the calculations, simulations and graphics shown in this paper can be accessed at: https://github.com/kaiu85/CRNs (accessed on 26 August 2021).
